# Plasma-Depleted Lyophilized Porcine Platelet Lysate as an Alternative to Fetal Bovine Serum in Cell Culture

**DOI:** 10.3390/life15121915

**Published:** 2025-12-14

**Authors:** Kuo-Chung Cheng, Hung-Maan Lee, Yi-Ting Shu, Yi-Chieh Chu, Jui-Ting Hsiao, Ming-Fa Hsieh, Yi-Ho Hsieh

**Affiliations:** 1 Department of Orthopedics, Taoyuan Armed Forces General Hospital, No. 168, Zhongxing Rd., Longtan Dist., Taoyuan City 325208, Taiwan; chichino183@hotmail.com (K.-C.C.); hungmaan@ms12.hinet.net (H.-M.L.); 2 Department of Materials and Textiles, Asia Eastern University of Science and Technology, No. 58, Sec. 2, Sihchuan Rd., Banqiao Dist., New Taipei City 220303, Taiwan; fc020@mail.aeust.edu.tw; 3 Department of Orthopedics, Min-Sheng General Hospital, No. 168, Jingguo Rd., Taoyuan Dist., Taoyuan City 330056, Taiwan; lala19930106@gmail.com (Y.-C.C.); bj22001@outlook.com (J.-T.H.); 4 Department of Biomedical Engineering, Chung Yuan Christian University, No. 200, Zhongbei Rd., Zhongli Dist., Taoyuan City 320314, Taiwan; mfhsieh@cycu.edu.tw; 5 School of Medicine, Tzu Chi University, No. 701, Sec. 3, Zhongyang Rd., Hualien City 970374, Taiwan

**Keywords:** plasma-depleted lyophilized porcine platelet lysate (pPL), cell culture, fetal bovine serum alternative, growth factors

## Abstract

Purpose: Fetal bovine serum (FBS) is widely used in cell culture due to its rich nutrient and growth factor content, but it poses ethical concerns, biosafety risks, and cost limitations. This study investigates plasma-depleted lyophilized (freeze-dried) porcine platelet lysate (pPL) as a potential alternative to FBS for use in cell-based research and biomanufacturing. Materials and Methods: Fresh porcine blood was processed to obtain plasma-depleted pPL using double centrifugation and repeated freeze–thaw cycles. The lysate was analyzed for growth factor content via ELISA, then freeze-dried and sterilized with gamma irradiation. Endotoxin levels and cytotoxicity were evaluated. The ability of plasma-depleted lyophilized pPL to promote cell proliferation was assessed using L929 fibroblast cultures and compared with FBS. Results: Plasma-depleted lyophilized pPL contained significantly higher levels of TGF-β1 than FBS. The freeze-dried product remained stable for at least three months at room temperature. Gamma irradiation effectively sterilized the lysate without degrading key growth factors. Plasma-depleted lyophilized pPL showed no cytotoxicity and promoted greater proliferation of L929 cells compared to FBS, indicating enhanced mitogenic activity. Conclusions: Plasma-depleted lyophilized pPL is a stable, safe, and growth factor-rich alternative to FBS. It supports fibroblast proliferation, retains bioactivity after sterilization and storage, and may provide a scalable, ethical option for cell culture in biomedical research, regenerative medicine, and therapeutic product development.

## 1. Introduction

Cell culture is an essential tool in biomedical research, tissue engineering, drug development, and regenerative medicine. Although fetal bovine serum (FBS) is widely used due to its rich content of growth factors, hormones, and nutrients, it raises major ethical, scientific, and practical concerns. FBS is obtained by cardiac puncture of bovine fetuses, raising unresolved animal welfare issues. Its use is also associated with batch-to-batch variability, risk of zoonotic transmission (e.g., prions, bovine viral diarrhea virus), and high cost, all of which limit its reliability and sustainability in research and clinical applications [1,2].

Numerous attempts have been made to develop serum-free or chemically defined media for specific cell types, yet no universal replacement has emerged [3,4,5]. Human platelet lysate (hPL) has emerged as a promising alternative, offering a xeno-free, human-derived supplement rich in multiple growth factors, including platelet-derived growth factor (PDGF), transforming growth factor-β1 (TGF-β1), epidermal growth factor (EGF), and vascular endothelial growth factor (VEGF) [6,7]. However, hPL faces its own challenges, including limited supply, donor variability, disease-transmission risks, and dependence on cold-chain logistics. These limitations highlight the need for alternative serum supplements with improved stability, safety, and scalability.

To address these challenges, porcine-derived products have been considered due to their availability and strong biological similarity to human proteins. Porcine blood—an abundant agricultural by-product—offers a sustainable resource for developing alternatives to FBS. While porcine-derived biomaterials are widely accepted in medical devices, the potential of porcine platelet lysate (pPL) in cell culture remains underexplored. Freeze-dried formulations in particular may provide advantages in long-term storage, transport simplicity, and cost-effectiveness.

In our previous experiments, we developed porcine platelet lysate (pPL) for the treatment of arthritis and wound healing in rabbits and rats, achieving positive therapeutic outcomes [8,9]. Related studies also support the use of pPL to replace fetal bovine serum to enhance cell proliferation, maintain differentiation potential, and ensure immune phenotype stability [10,11]. However, when using pPL as a substitute for fetal bovine serum (FBS) in cell culture, several issues arose that affected the results. First, the presence of plasma components in porcine platelet lysate led to the formation of fibrin gels, which interfered with cell distribution. Although anticoagulants can prevent fibrin formation, excessive use of anticoagulants may also inhibit cell growth. Furthermore, adult porcine plasma used as a supplement in cell culture contains myriad plasma proteins, immunoglobulins, lipids and other low-molecular-weight bioactive substances, many of which remain undefined; hence, the system inherits additional batch-to-batch variability and intrinsic uncertainty in cell response [12,13]. This is why fetal bovine serum is preferred over adult bovine serum; it contains fewer undefined or variable components that may influence cell behavior [1,14]. Therefore, we designed further experiments to remove plasma components and retain only platelet-derived growth factors, aiming to utilize these growth factors for cell culture while eliminating the adverse effects associated with plasma.

Building on these considerations, this study focuses on developing plasma-depleted lyophilized pPL by isolating porcine platelets, applying controlled freeze–thaw cycles for lysis, removing plasma components, and performing gamma sterilization. The lyophilized format enables long-term stability at ambient temperature and avoids the need for refrigeration. To evaluate its suitability as a serum substitute, we conducted growth factor analysis, endotoxin and sterility testing, cytotoxicity assessment, and fibroblast proliferation assays using L929 cells.

Our overarching aim is to determine whether plasma-depleted lyophilized pPL can serve as a cost-effective, ethically acceptable, and functionally robust alternative to FBS. The use of agricultural by-products aligns with global sustainability trends while reducing reliance on fetal-derived materials. Successful development of such a xenogeneic, standardized platform could advance both laboratory-scale research and clinical biomanufacturing, providing a stable and ethically sound pathway toward next-generation serum alternatives.

## 2. Materials and Methods

### 2.1. Preparation of Plasma-Depleted Lyophilized pPL

Fresh whole blood (40 mL) was obtained from healthy pigs (age 5–6 months) under hygienic and controlled conditions from a licensed abattoir. Blood was collected into acid-citrate-dextrose (ACD) tubes to prevent coagulation. The initial centrifugation was performed at 1000× *g* for 5 min at 20 °C to remove red blood cells. The supernatant was carefully collected and filtered through a leukocyte reduction filter (SAFETRAN Leukocyte Reduction Filter/Transfusion set) to minimize immunogenic impurities. Platelets were pelleted by centrifugation at 2000× *g* for 20 min. The plasma was removed, and the resulting platelet pellet was resuspended in calcium- and magnesium-free phosphate-buffered saline (PBS) to minimize potential plasma-derived interference. Platelet lysis was achieved through three repeated freeze–thaw cycles at −80 °C and 37 °C. Post-lysis, lysates were centrifuged at 2000× *g* for 20 min to eliminate residual debris. The supernatant was then filtered through a 0.22 μm PES filter and lyophilized using a laboratory freeze-dryer (−55 °C condenser, <100 mTorr) for 24 h. The resulting powder was stored at room temperature in sterilized vials under dry conditions until further analysis.

### 2.2. Quantification of Growth Factors

Platelets contain a wide range of growth factors, among which transforming growth factor beta 1 (TGF-β1) was selected as a representative marker for analysis. The concentration of TGF-β1 was quantified using a commercial ELISA kit (R&D Systems, Minneapolis, MN, USA). Prior to analysis, lyophilized pPL was reconstituted in ultrapure water and diluted 1:5 with assay buffer. ELISA was performed according to manufacturer instructions, with standards and samples loaded in duplicate onto pre-coated 96-well plates. Absorbance was measured at 450 nm with wavelength correction at 540 nm using a microplate reader. Standard curves were generated from known concentrations of recombinant TGF-β1 to interpolate sample values. For comparison, FBS samples from three commercial lots were analyzed under identical conditions.

### 2.3. Stability of Plasma-Depleted Lyophilized pPL at Room Temperature

Aliquots of plasma-depleted lyophilized pPL were stored at ambient temperature (18–20 °C) in sealed vials protected from light and humidity. At intervals of 0, 30, 60, and 90 days, samples were reconstituted and analyzed for TGF-β1 concentration by ELISA. Physical properties including powder color, solubility, and reconstitution clarity were recorded. The recovery percentage was calculated relative to baseline concentrations. A coefficient of variation (CV) < 10% was considered acceptable for reproducibility.

### 2.4. Gamma Irradiation Sterilization and Sterility Test

Plasma-depleted lyophilized pPL samples were sterilized by gamma irradiation (25–35 kGy) using a cobalt-60 source at a certified facility of the Atomic Energy Council (New Taipei City, Taiwan). To assess sterility, 1 mL reconstituted plasma-depleted lyophilized pPL was inoculated into 10 mL of tryptic soy broth (TSB) and incubated at 37 °C for 14 days. Tubes were examined daily for turbidity. Additionally, samples were plated on blood agar to detect bacterial and fungal contamination. Pre- and post-irradiation TGF-β1 levels were measured to evaluate the impact on growth factor stability.

### 2.5. Cytotoxicity Assessment by MTT Assay

Cytotoxicity was evaluated using the MTT (3-(4,5-dimethylthiazol-2-yl)-2,5-diphenyltetrazolium bromide) assay according to ISO 10993-5 standards [15]. using L929 fibroblast cells. Cells were seeded at 5 × 10^3^ cells/well in 96-well plates and allowed to adhere overnight. Media were replaced with EMEM supplemented with 10% reconstituted plasma-depleted pPL or FBS. After 24 h, 20 µL of MTT solution (5 mg/mL) was added to each well and incubated for 4 h at 37 °C. Formazan crystals were solubilized in DMSO, and absorbance was measured at 570 nm. Viability was calculated as a percentage of untreated controls. Triplicate samples were analyzed in three independent experiments.

### 2.6. Endotoxin Testing

Endotoxin content was assessed using the gel-clot Limulus Amebocyte Lysate (LAL) assay (Lonza, Portsmouth, NH, USA). Reconstituted plasma-depleted lyophilized pPL samples (100 µL) were mixed with equal volumes of LAL reagent and incubated at 37 °C for 1 h. A positive result was defined as firm gel formation that remained intact upon inversion. All tests were performed in duplicate with included positive and negative controls. The sensitivity of the assay was 0.25 EU/mL, where EU/mL denotes endotoxin units per milliliter as defined by the U.S. Pharmacopeia.

### 2.7. Cell Proliferation Assay (L929 Cells)

To evaluate mitogenic effects, L929 cells were seeded at 5000 cells/well in 24-well plates and cultured in EMEM with 10% plasma-depleted lyophilized pPL or FBS. Cells were trypsinized and counted daily for three days using trypan blue exclusion. Data were plotted as growth curves. Additional proliferation was assessed using a resazurin-based assay (PrestoBlue) for fluorescence-based quantification of metabolic activity.

L929 murine fibroblasts were selected because they represent one of the most widely used and internationally accepted cell lines for cytotoxicity and biocompatibility evaluations, including assays based on ISO 10993-5. Their stable growth characteristics, high reproducibility, and sensitivity to impurities make them an appropriate first-line model for assessing the biological activity and safety of serum substitutes. Using L929 cells ensures standardized comparison with existing literature and provides a consistent baseline for evaluating the proliferative effects of plasma-depleted lyophilized pPL.

### 2.8. Statistical Analysis

All quantitative data are expressed as the mean ± standard error (SE). Each experiment was independently repeated three times (biological replicates = 3), and each sample was measured in duplicate (technical replicates = 2). Comparisons between two groups were evaluated using Student’s *t*-test, while multiple group comparisons were performed using one-way ANOVA. Differences were considered statistically significant at *p* < 0.05.

## 3. Results

### 3.1. Efficient Preparation and Quality of Plasma-Depleted Lyophilized pPL

**Figure 1:**
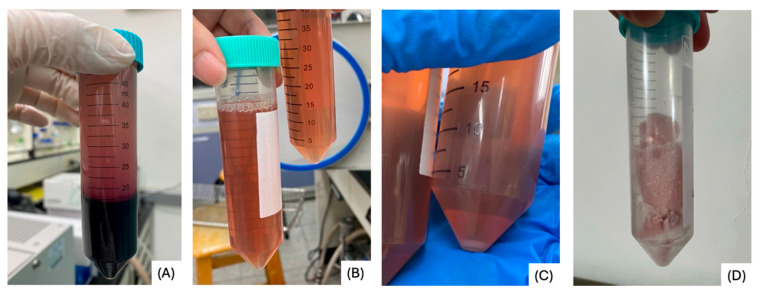
Preparation process of plasma-depleted lyophilized pPL. This schematic illustrates the centrifugation, leukocyte filtration, platelet lysis, and freeze-drying steps used to generate plasma-depleted lyophilized pPL. (**A**) Porcine whole blood after the first centrifugation, showing separation of red blood cells. (**B**) Plasma after leukocyte filtration to remove white blood cells. (**C**) Concentrated platelet pellet obtained after the second centrifugation. (**D**) Plasma-depleted lyophilized pPL was produced following repeated freeze–thaw cycles and vacuum freeze-drying.

Plasma-depleted lyophilized pPL was successfully produced using a reproducible centrifugation and filtration protocol. Initial centrifugation removed red blood cells, and leukocyte filtration eliminated most white blood cells, ensuring reduced immunogenicity. The second centrifugation step formed a platelet pellet; the platelet pellet was resuspended in 1 mL PBS. The platelet count increasing from 1.62 × 10^5^/µL in whole blood to 5.49 × 10^5^/µL. After three freeze–thaw cycles, platelets were effectively lysed, decreasing count to 0.4 × 10^5^/µL, indicative of efficient release of intracellular granules. The final product, a sterile lyophilized powder, was easy to reconstitute, demonstrated good solubility, and had no visible particulate matter or discoloration. These qualities make it a favorable candidate for use as a serum alternative in clinical and research applications (Figure 1).

### 3.2. Plasma-Depleted Lyophilized pPL Contains Higher TGF-β1 Concentration than FBS

**Figure 2:**
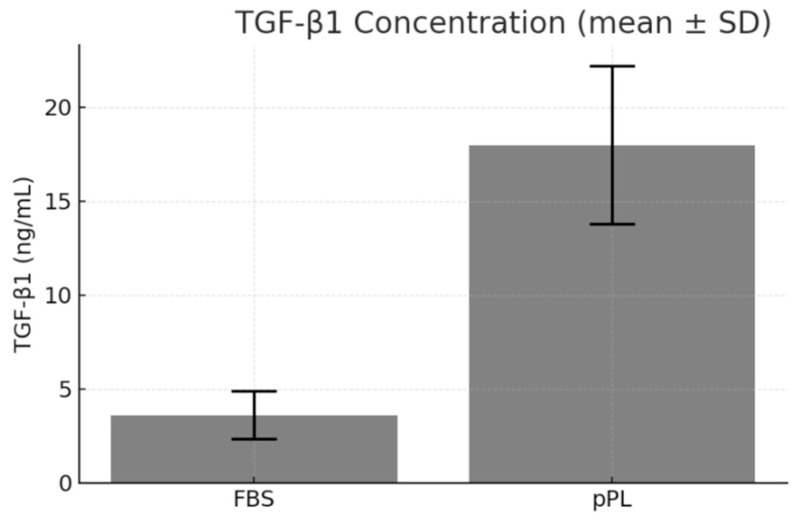
Comparison of growth factor concentrations between plasma-depleted lyophilized porcine platelet lysate (pPL) and fetal bovine serum (FBS). The concentration of TGF-β1, used as a representative marker, was markedly higher in plasma-depleted lyophilized pPL compared with FBS, indicating a richer growth factor profile in pPL. Data are expressed as mean ± SD from three independent experiments (*n* = 3).

Quantitative ELISA assays revealed that TGF-β1 levels in plasma-depleted lyophilized pPL (18.26 ng/mL) were significantly higher than in standard FBS (4.06 ng/mL), representing a 4.5-fold increase (*p* < 0.01) (Figure 2). These results were consistent across three independently prepared plasma-depleted lyophilized pPL batches, indicating high reproducibility. The elevated TGF-β1 concentration supports the hypothesis that porcine-derived platelets can serve as a potent reservoir of growth factors critical for cellular expansion and differentiation.

### 3.3. Stability of Growth Factors in Plasma-Depleted Lyophilized pPL at Room Temperature

**Figure 3:**
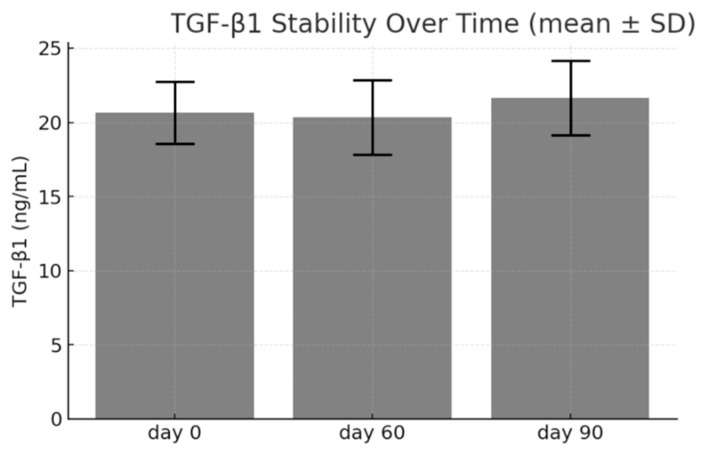
The plasma-depleted lyophilized pPL demonstrated excellent storage stability under ambient conditions. Quantitative analysis of TGF-β1 content revealed minimal variation throughout 90 days of storage (*p* > 0.05), indicating that plasma-depleted lyophilization effectively preserves the bioactivity and structural integrity of growth factors. These findings support the suitability of plasma-depleted lyophilized pPL for long-term storage and transportation without the need for cold-chain logistics. Data are expressed as mean ± SD from three independent experiments (*n* = 3).

Reconstituted lyophilized pPL was assessed after storage at ambient temperature (18–20 °C) for 0, 30, 60, and 90 days. TGF-β1 concentration remained within 95–105% of the initial value across all timepoints with no statistically significant difference (ANOVA, *p* > 0.05) (Figure 3). The plasma-depleted lyophilized powder retained solubility and clarity after each storage interval. These findings suggest that the plasma-depleted lyophilized format is not only convenient for transport and storage but also maintains the integrity of the key bioactive components over time. Further tests at 6- and 12-month intervals are planned to define the full shelf-life.

### 3.4. Gamma Sterilization Does Not Affect Growth Factor Concentration

**Figure 4:**
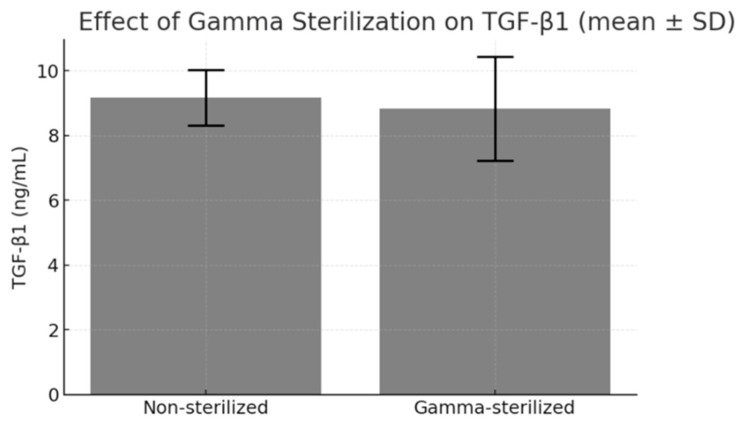
Impact of gamma irradiation on TGF-β1 concentration. TGF-β1 levels in plasma-depleted lyophilized pPL remained stable following gamma sterilization at 25–35 kGy, showing no statistically significant reduction compared with non-sterilized samples (*p* > 0.05). Data are expressed as mean ± SD from three independent experiments (*n* = 3).

Gamma irradiation at 25–35 kGy was used to sterilize the plasma-depleted lyophilized pPL. ELISA results indicated no significant change in TGF-β1 concentration before and after irradiation (*p* > 0.05) (Figure 4). Sterility tests in TSB showed no turbidity or microbial growth over 14 days in any of the 10 replicates, validating sterilization efficacy. Morphological inspection under light microscopy revealed no visible aggregates or structural degradation in the reconstituted powder post-irradiation. These findings confirm that gamma irradiation is suitable for the terminal sterilization of plasma-depleted lyophilized pPL without compromising its growth factor concentration.

### 3.5. Cytocompatibility Confirmed by MTT Assay

**Figure 5:**
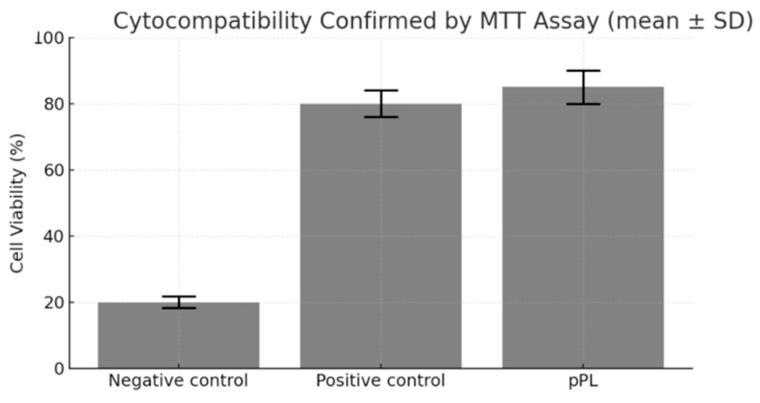
Cytotoxicity evaluation using MTT assay. Cell viability exceeded 85% in culture media supplemented with plasma-depleted lyophilized pPL, comparable to that of the positive control group. This result indicates that plasma-depleted lyophilized pPL exhibited no detectable cytotoxicity under the tested conditions, supporting its excellent biocompatibility and suitability for further biomedical applications. Data are expressed as mean ± SD from three independent experiments (biological replicates = 3, technical replicates = 2).

L929 fibroblasts cultured in medium supplemented with 10% plasma-depleted lyophilized pPL exhibited viability exceeding 85% across three independent replicates, compared to 80% in the FBS group (*p* > 0.05) (Figure 5). Cell morphology remained intact, with typical spindle-shaped fibroblast appearance maintained throughout 24-h culture. These results demonstrate that plasma-depleted lyophilized pPL is non-cytotoxic and compatible with mammalian cell lines. Additional tests with endothelial and epithelial cell lines are planned to broaden safety validation.

### 3.6. Endotoxin Testing Confirms Safety of Plasma-Depleted Lyophilized pPL

LAL gel-clot assays showed no endotoxin presence in plasma-depleted lyophilized pPL samples. Positive controls exhibited gel formation, whereas all test replicates and negative controls remained clear, indicating endotoxin levels below detection thresholds (<0.25 EU/mL). This supports the suitability of the preparation process for clinical research applications requiring endotoxin-free conditions.

### 3.7. Plasma-Depleted Lyophilized pPL Enhances Fibroblast Proliferation Compared to FBS

**Figure 6:**
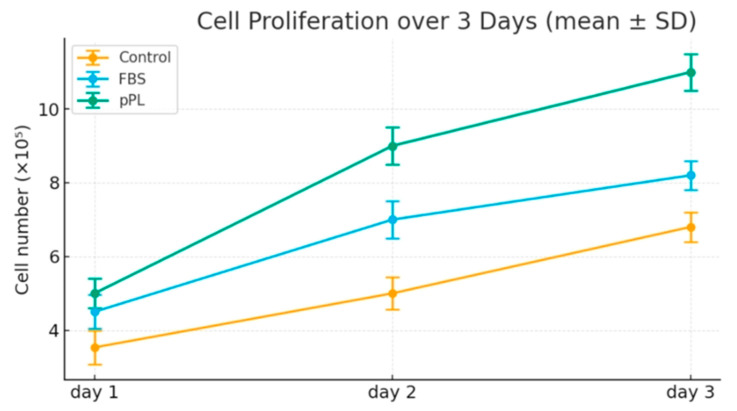
Proliferation of L929 cells with plasma-depleted lyophilized pPL vs. FBS. Plasma-depleted lyophilized pPL supplementation significantly enhanced cell growth compared with FBS and control groups over 3 days (*p* < 0.05), demonstrating stronger mitogenic activity and superior serum replacement potential. Data are expressed as mean ± SD from three independent experiments (*n* = 3).

L929 fibroblast proliferation was significantly enhanced in the plasma-depleted lyophilized pPL group over 3 days. Day 1, 2, and 3 cell counts were 18%, 26%, and 34% higher than the FBS group (*p* < 0.05 at all timepoints). Growth curves derived from repeated cell counts confirmed a steeper slope in the plasma-depleted lyophilized pPL group, suggesting stronger mitogenic activity. These findings support the hypothesis that growth factor-enriched plasma-depleted lyophilized pPL can outperform traditional FBS in promoting rapid cell expansion (Figure 6).

## 4. Discussion

With the continuous expansion of cell technology and increasing attention to animal welfare, finding a reliable, ethical, and effective alternative to FBS has become a major focus in the field of cell culture. This study investigated the feasibility of using plasma-depleted lyophilized pPL, prepared from slaughterhouse-derived whole porcine blood, as a serum substitute for cell culture. The results demonstrated that plasma-depleted lyophilized pPL not only performs comparably to FBS but even surpasses it in promoting cell proliferation.

Plasma-depleted lyophilized pPL contains a variety of growth factors, with TGF-β1 analyzed as a representative marker. One of the key findings of this study is that the concentration of TGF-β1 in plasma-depleted lyophilized pPL is markedly higher than that in FBS, suggesting a concurrent enrichment of other growth factors as well. Since growth factors play crucial roles in regulating cell proliferation, extracellular matrix synthesis, and differentiation, it is reasonable to infer that their elevated levels are closely associated with the enhanced proliferation observed in L929 fibroblasts.

In addition to its biological potency, the lyophilized formulation offers practical advantages. Lyophilization not only facilitates long-term storage and transportation but also allows flexible reconstitution at desired concentrations according to experimental or clinical needs. This adjustability enables researchers to fine-tune the concentration of bioactive molecules for specific cell types or regenerative applications, thereby improving both reproducibility and scalability. Furthermore, plasma-depleted lyophilized pPL demonstrates excellent stability and sustained bioactivity, underscoring its potential as a robust serum substitute. Pan et al. (2016) reported that lyophilized porcine platelet-rich plasma maintained stable levels of key growth factors, including PDGF, TGF-β, and VEGF, even after four weeks of storage, indicating that the lyophilization process effectively preserves platelet-derived bioactive molecules over time [16,17]. Consistent with these findings, our data show that plasma-depleted lyophilized pPL can be stored at room temperature for at least three months without measurable degradation of growth factor integrity.

Additionally, gamma irradiation at 25–35 kGy provided effective terminal sterilization while maintaining the biological activity of major growth factors. This observation aligns with the reports by Ma et al. (2018), who demonstrated that gamma-irradiated human platelet lysate retained comparable growth-promoting efficacy for mesenchymal stem cell expansion, and by Shukla et al. (2016), who found that irradiation caused only minimal alterations in platelet-derived cytokine and growth factor release profiles [18,19]. Finally, MTT cytotoxicity and LAL endotoxin assays verified that the plasma-depleted lyophilized pPL formulation is non-toxic and endotoxin-free, highlighting its safety, stability, and suitability as a scalable culture supplement for cell-based biomanufacturing.

Unlike FBS, which often exhibits significant batch-to-batch variation in growth factor composition and performance, plasma-depleted lyophilized pPL offers chemical consistency and high reproducibility. This characteristic not only enhances experimental reproducibility but also facilitates standardization across laboratories and production lots. In the future, customized formulations containing specific ratios of growth factors could be developed to meet the proliferation or differentiation requirements of different cell types. Such tailored control of the cellular microenvironment could benefit fields including basic cell biology, regenerative medicine, and large-scale cell manufacturing, highlighting plasma-depleted pPL’s strong potential as a standardized serum substitute.

From a biological perspective, the enhanced cell-growth effect of plasma-depleted pPL may result from the synergistic release of multiple growth factors during platelet lysis. Platelets are known to store and release multiple growth factors that collectively promote cell proliferation, matrix remodeling, and tissue repair [20,21]. Although this study primarily focused on TGF-β1 as a representative biomarker, future studies should include quantitative analyses of additional growth factors—such as PDGF, VEGF, and IGF-1—to better elucidate their combined effects on cell behavior and to provide a more comprehensive understanding of the bioactive profile of plasma-depleted lyophilized pPL.

Despite the promising results, several limitations remain. First, although gamma irradiation did not alter the concentration of TGF-β1, its potential impact on protein conformation or bioactivity requires further verification through functional assays, such as Smad phosphorylation or downstream gene expression analysis [22,23]. Ionizing radiation can induce structural modifications in protein molecules, including partial unfolding or oxidation of amino acid residues, which may compromise receptor binding and signaling efficacy. However, many reports have demonstrated that when irradiation is performed under optimized and controlled conditions, protein structural stability and functional activity can be well preserved. For instance, Smeltzer et al. (2015) showed that plasma-derived IgG maintained its tertiary structure and antigen-binding activity after exposure to gamma doses up to 35 kGy [24], while Ordoyo-Pascual et al. (2025) reported negligible alterations in growth factor profiles following beta and gamma sterilization [25]. Therefore, confirming the preservation of TGF-β1 functionality after gamma sterilization would be helpful to ensure the biological reliability of irradiated pPL formulations. Second, this study used mouse fibroblasts as a compatibility model; future studies should extend to human cells and stem cells to evaluate cross-species applicability and scalability. Third, although no endotoxin was detected, large-scale manufacturing will still require rigorous quality control and standardization protocols.

In previous approaches utilizing FBS or plasma-containing platelet lysate, a variety of plasma proteins and fibrin formation frequently interfered with cell culture performance and reproducibility. These plasma-derived components can lead to unwanted gel formation, heterogeneous cell distribution, or altered nutrient diffusion within the culture system, ultimately influencing experimental outcomes and complicating standardization across batches. To mitigate these effects, multiple technical modifications have been developed, including enzymatic or calcium/thrombin-induced defibrinogenation to remove fibrin precursors, ultrafiltration to eliminate high-molecular-weight plasma proteins while retaining low-molecular-weight growth factors, and chromatographic purification to selectively enrich mitogenic factors and deplete immunogenic or pro-inflammatory proteins [26,27].

In contrast, the Plasma-depleted lyophilized pPL formulation adopted in this study effectively eliminates fibrinogen and plasma-derived proteins during the preparation process itself, providing a cleaner, heparin-free supplement that supports consistent and reproducible cell growth. This intrinsic purification step obviates the need for additional processing or chemical additives and minimizes batch variability while maintaining the native profile of platelet-derived growth factors. Consequently, plasma-depleted pPL represents an optimized and simplified alternative to conventional PL or FBS supplements, offering a more physiologically relevant yet experimentally stable culture environment suitable for translational and clinical applications.

In cross-species applications, potential immunogenicity and the risk of xenogeneic pathogen transmission must be carefully considered. In this study, we minimized immunogenic components by depleting plasma, removing leukocytes, and lysing platelets to retain primarily intracellular growth factors while eliminating most plasma proteins and cellular antigens, as detailed in our previous work [8]. This preparation strategy significantly reduces the likelihood of immune activation compared with conventional plasma-containing platelet lysates. Moreover, terminal sterilization using 25–35 kGy gamma irradiation provides effective bacterial sterilization and viral inactivation, offering an additional safety layer. Nonetheless, although gamma irradiation is recognized for its broad-spectrum virucidal effect, comprehensive viral reduction testing targeting specific porcine viruses (e.g., circoviruses, parvoviruses, and enveloped vs. non-enveloped viruses) will be required in future studies to fully establish the biosafety profile of plasma-depleted lyophilized pPL for translational use.

Numerous studies have compared the molecular structures and biological activities of porcine and human growth factors, demonstrating a high degree of sequence homology and functional conservation across species. For instance, porcine and human TGF-β exhibit nearly identical ligand-receptor binding and activation properties; early receptor-binding assays using radiolabeled ^125^I-porcine and ^125^I-human TGF-β revealed no significant difference in receptor affinity, indicating conserved engagement of the TGF-β/Smad signaling axis between the two species [28]. Similarly, porcine platelet-derived PDGF displayed receptor-binding kinetics virtually indistinguishable from those of human PDGF when assayed against human placental membrane receptors [29]. In addition, porcine epidermal growth factor (EGF) isolated from uterine tissue shares substantial structural similarity with human EGF and exhibits comparable receptor-mediated mitogenic activity [30]. Moreover, porcine insulin-like growth factor-I (IGF-I) possesses an amino acid sequence nearly identical to that of human IGF-I and shows cross-reactivity in human receptor and immunoassays [31]. Collectively, these findings provide strong evidence of cross-species structural and functional equivalence among major porcine and human growth factors, supporting the scientific rationale for developing plasma-depleted lyophilized pPL as a biologically compatible and scalable alternative to human platelet lysate in biomedical and cell-culture applications.

In conclusion, this study highlights the scientific significance and industrial potential of plasma-depleted lyophilized pPL. It combines the biological activity of platelet-derived growth factors with the practical advantages of a stable powder formulation. Through further process optimization, plasma-depleted lyophilized pPL could emerge as a stable, low-cost, and clinically scalable alternative to FBS. Developing supplements based on plasma-depleted lyophilized pPL not only represents a major advancement in replacing FBS but also embodies a sustainable innovation pathway, transforming agricultural by-products into valuable resources for regenerative medicine.

## 5. Conclusions

This study demonstrates that plasma-depleted lyophilized pPL is a viable and superior alternative to fetal bovine serum in cell culture. Plasma-depleted lyophilized pPL is rich in biologically active growth factors and supports robust cell proliferation. The product is stable at room temperature, amenable to gamma sterilization, free of cytotoxicity and endotoxins, and easily produced from a sustainable animal source. These findings underscore the potential of plasma-depleted lyophilized pPL as a next-generation culture supplement, addressing the ethical, logistical, and biological limitations of FBS. As global cell-based applications expand, the development of such animal by-product–derived materials could help bridge the gap between research and clinical translation, making regenerative therapies more accessible, reproducible, and ethically sound.

## Data Availability

The original contributions presented in this study are included in the article. Further inquiries can be directed to the corresponding author.
